# Supplementary material for: Refinement of High-Gamma EEG Features From TBI Patients With Hemicraniectomy Using an ICA Informed by Simulated Myoelectric Artifacts
Source: Front Neurosci. 2020 Nov 24;14:599010. doi: 10.3389/fnins.2020.599010 (PMC7732541; doi:10.3389/fnins.2020.599010)
Supplement: Supplementary file 1 [file Data_Sheet_1.PDF]

Part 2. Supplementary results

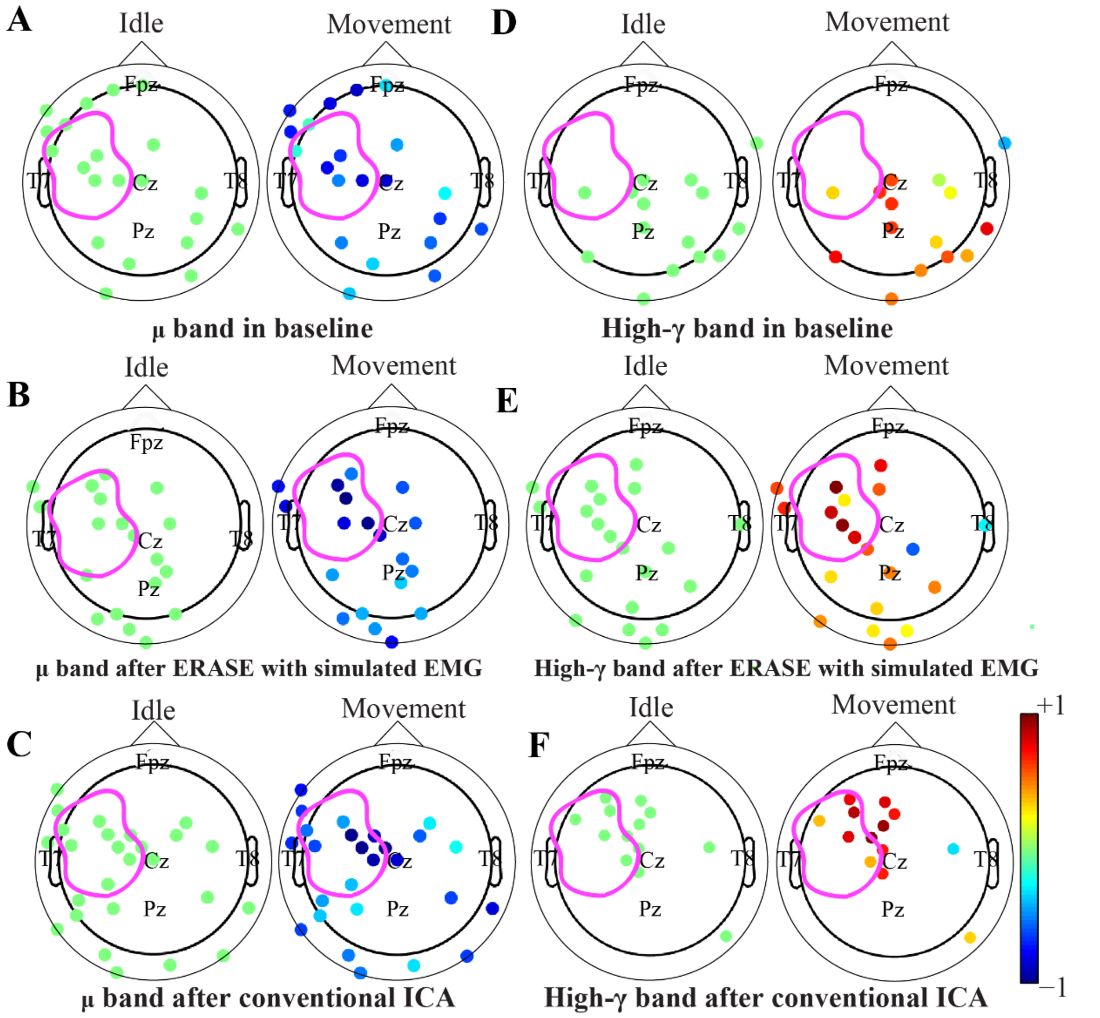

Fig. 1. Brain topography maps of z-scored μ and high-γ power before and after artifact rejection with ERASE with simulated EMG and conventional ICA on the Subject 1. Time for idle is 1 second before movement. Time for movement is 2 seconds. Only electrodes whose z-scored power of μ and high-γ during idle time and movement was significantly different were shown (Wilcoxon rank sum test). P-value for significant difference was 0.05. The dots outlined the position of the electrodes, and details of electrode position can refer to Fig. 11. Colours denoted the z-scored power of μ or high-γ in corresponding electrodes. The purple outline in each subfigure denoted the HA.

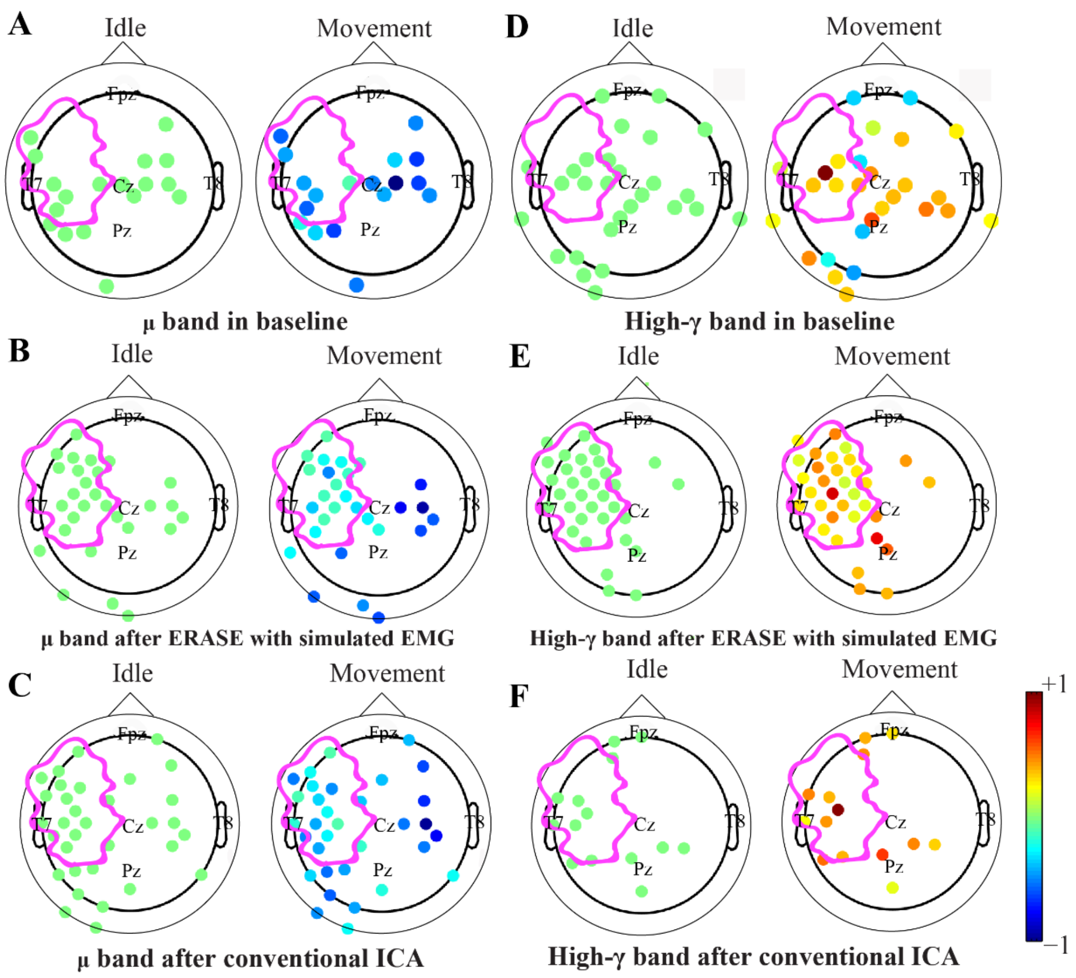

Fig. 2. Brain topography maps of z-scored μ and high-γ power before and after artifact rejection with ERASE with simulated EMG and conventional ICA on the Subject 2. Time for idle is 1 second before movement. Time for movement is 2 seconds. Only electrodes whose z-scored power of μ and high-γ during idle time and movement was significantly different were shown (Wilcoxon rank sum test). P-value for significant difference was 0.05. The dots outlined the position of the electrodes, and details of electrode position can refer to Fig. 11. Colours denoted the z-scored power of μ or high-γ in corresponding electrodes. The purple outline in each subfigure denoted the HA.

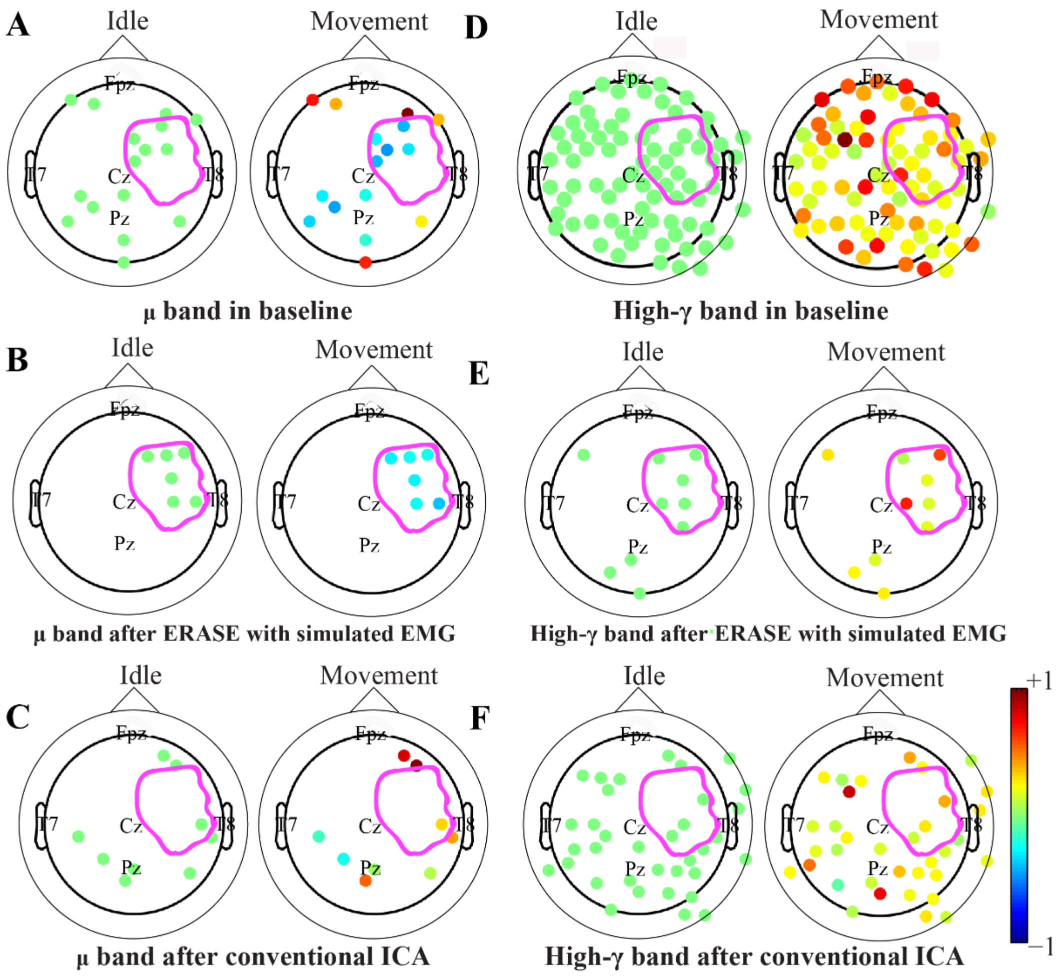

Fig. 3. Brain topography maps of z-scored μ and high-ᵞ power before and after artifact rejection with ERASE with simulated EMG and conventional ICA on the Subject 3. Time for idle is 1 second before movement. Time for movement is 2 seconds. Only electrodes whose z-scored power of μ and high-ᵞ during idle time and movement was significantly different were shown (Wilcoxon rank sum test). P-value for significant difference was 0.05. The dots outlined the position of the electrodes, and details of electrode position can refer to Fig. 11. Colours denoted the z-scored power of μ or high-ᵞ in corresponding electrodes. The purple outline in each subfigure denoted the HA.

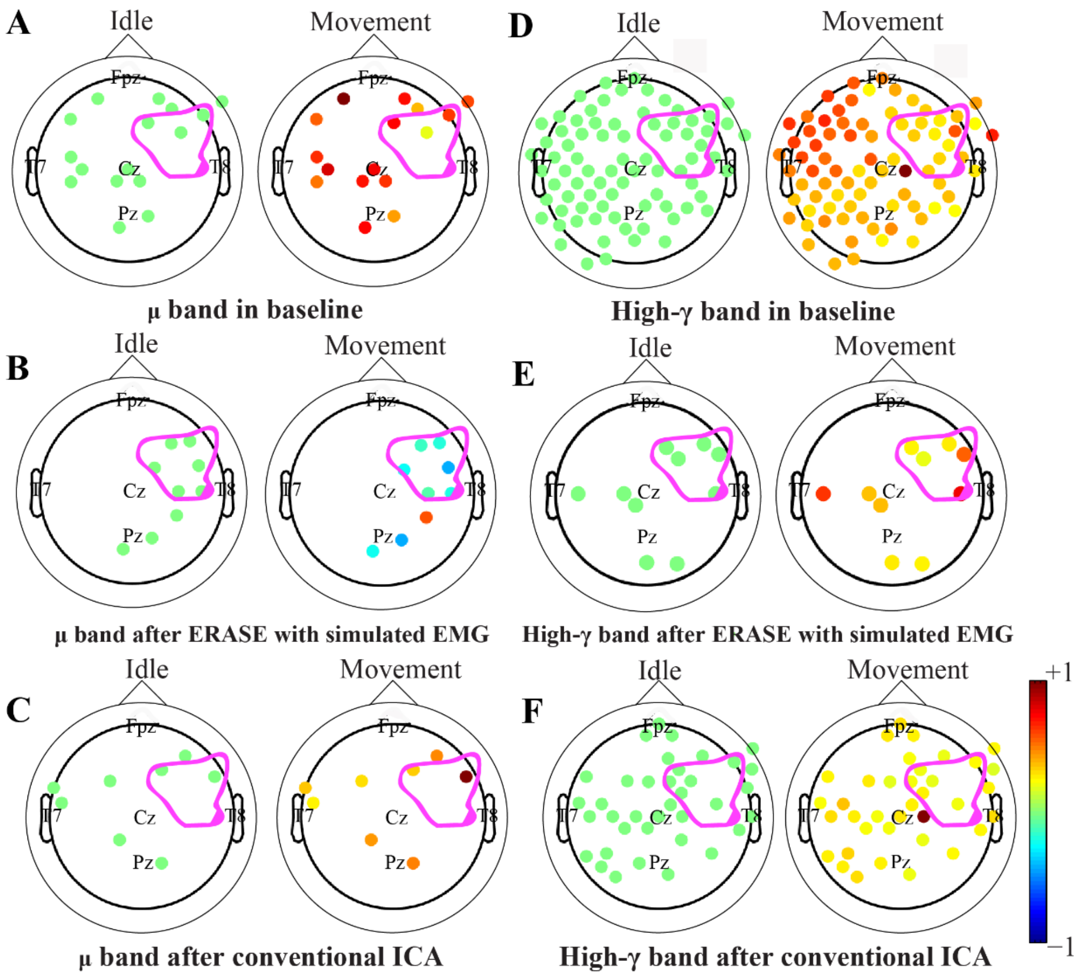

Fig. 4. Brain topography maps of z-scored μ and high-γ power before and after artifact rejection with ERASE with simulated EMG and conventional ICA on the Subject 4. Time for idle is 1 second before movement. Time for movement is 2 seconds. Only electrodes whose z-scored power of μ and high-γ during idle time and movement was significantly different were shown (Wilcoxon rank sum test). P-value for significant difference was 0.05. The dots outlined the position of the electrodes, and details of electrode position can refer to Fig. 11. Colours denoted the z-scored power of μ or high-γ in corresponding electrodes. The purple outline in each subfigure denoted the HA.

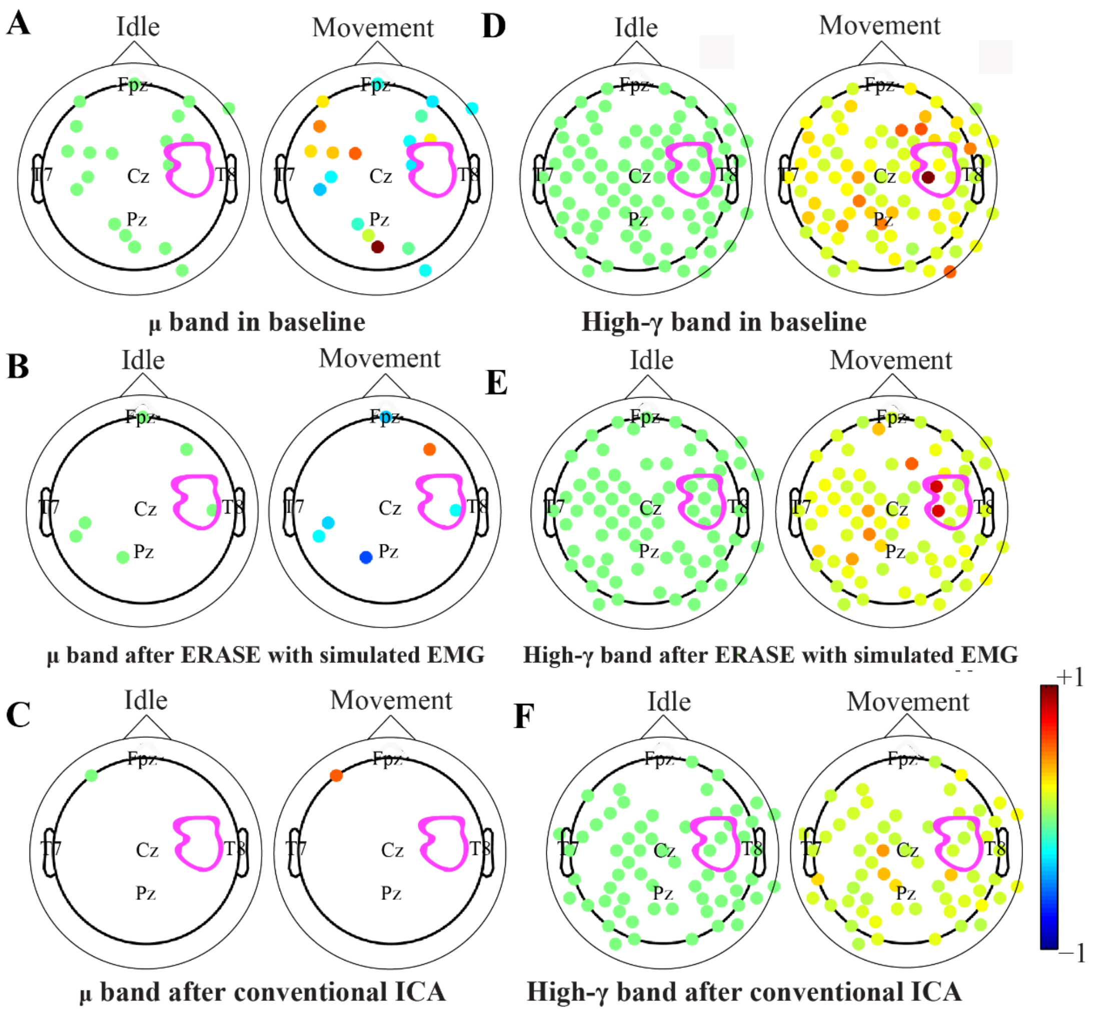

Fig. 5. Brain topography maps of z-scored μ and high-γ power before and after artifact rejection with ERASE with simulated EMG and conventional ICA on the Subject 5. Time for idle is 1 second before movement. Time for movement is 2 seconds. Only electrodes whose z-scored power of μ and high-γ during idle time and movement was significantly different were shown (Wilcoxon rank sum test). P-value for significant difference was 0.05. The dots outlined the position of the electrodes, and details of electrode position can refer to Fig. 11. Colours denoted the z-scored power of μ or high-γ in corresponding electrodes. The purple outline in each subfigure denoted the HA.

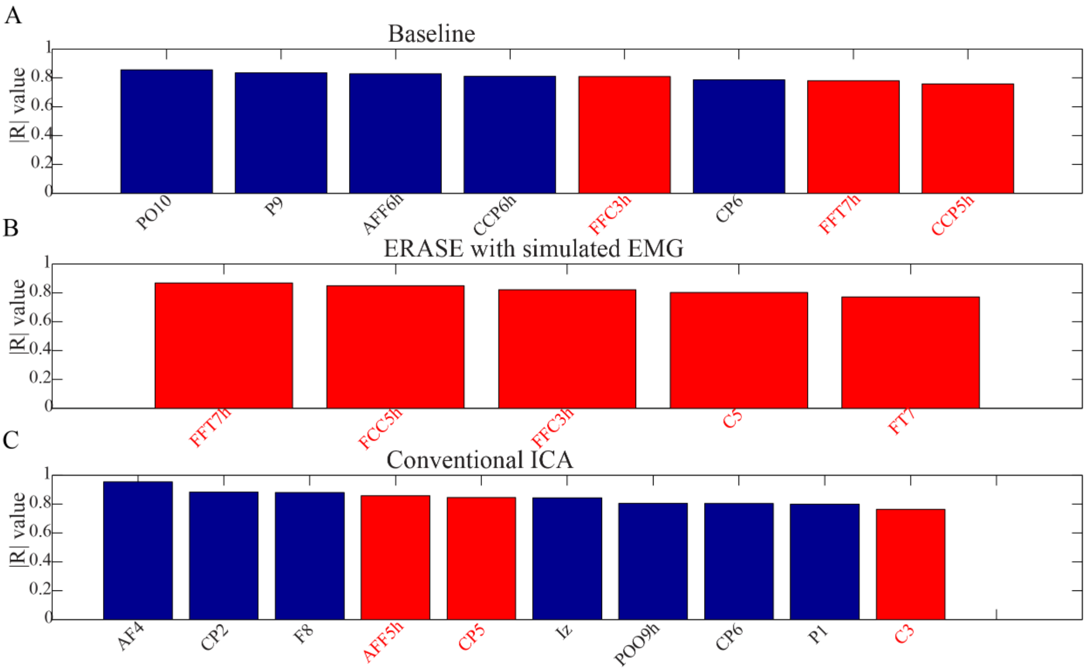

Fig. 6. Bar graphs for showing the electrodes with significant correlation in three conditions (baseline, after ERASE with simulated EMG and after conventional ICA). Here, the correlation coefficients were absolute values from 0 to 1. Data were from Subject 2. Blue bars denoted the electrodes with significant correlation in the NHAs, and red bars were the ones in HAs. A. the electrodes with significant correlation before ERASE. B. the electrodes with significant correlation after ERASE with simulated EMG. C. the electrodes with significant correlation after conventional ICA.

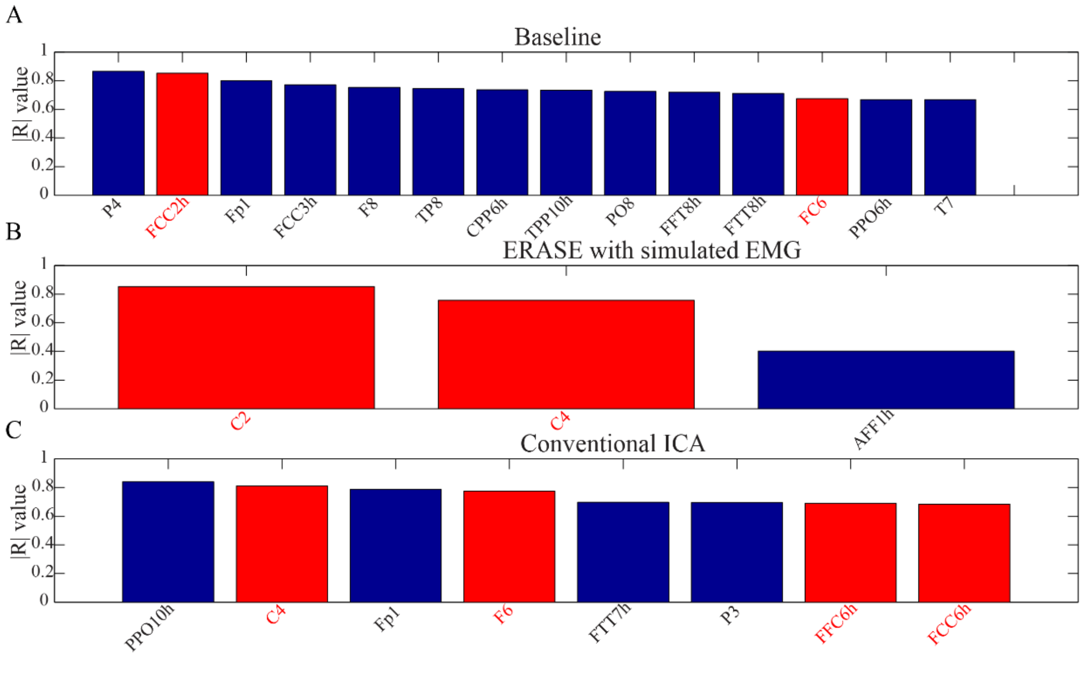

Fig. 7. Bar graphs for showing the electrodes with significant correlation in three conditions (baseline, after ERASE with simulated EMG and after conventional ICA). Here, the correlation coefficients were absolute values from 0 to 1. Data were from Subject 3. Blue bars denoted the electrodes with significant correlation in the NHAs, and red bars were the ones in HAs. A. the

electrodes with significant correlation before ERASE. B. the electrodes with significant correlation after ERASE with simulated EMG. C. the electrodes with significant correlation after conventional ICA.

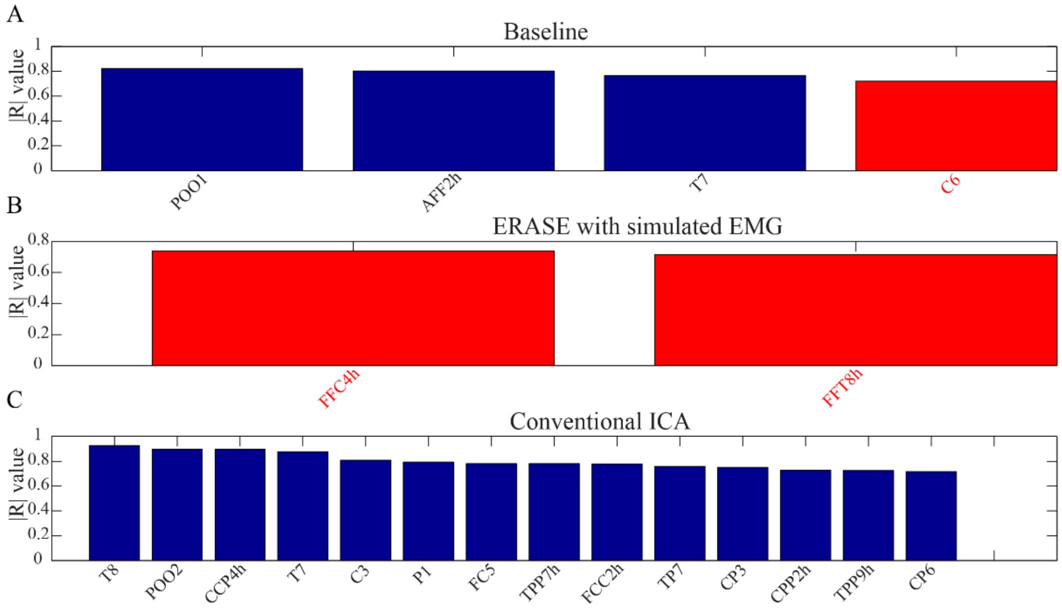

Fig. 8. Bar graphs for showing the electrodes with significant correlation in three conditions (baseline, after ERASE with simulated EMG and after conventional ICA). Here, the correlation coefficients were absolute values from 0 to 1. Data were from Subject 4. Blue bars denoted the electrodes with significant correlation in the NHAs, and red bars were the ones in HAs. A. the electrodes with significant correlation before ERASE. B. the electrodes with significant correlation after ERASE with simulated EMG. C. the electrodes with significant correlation after conventional ICA.

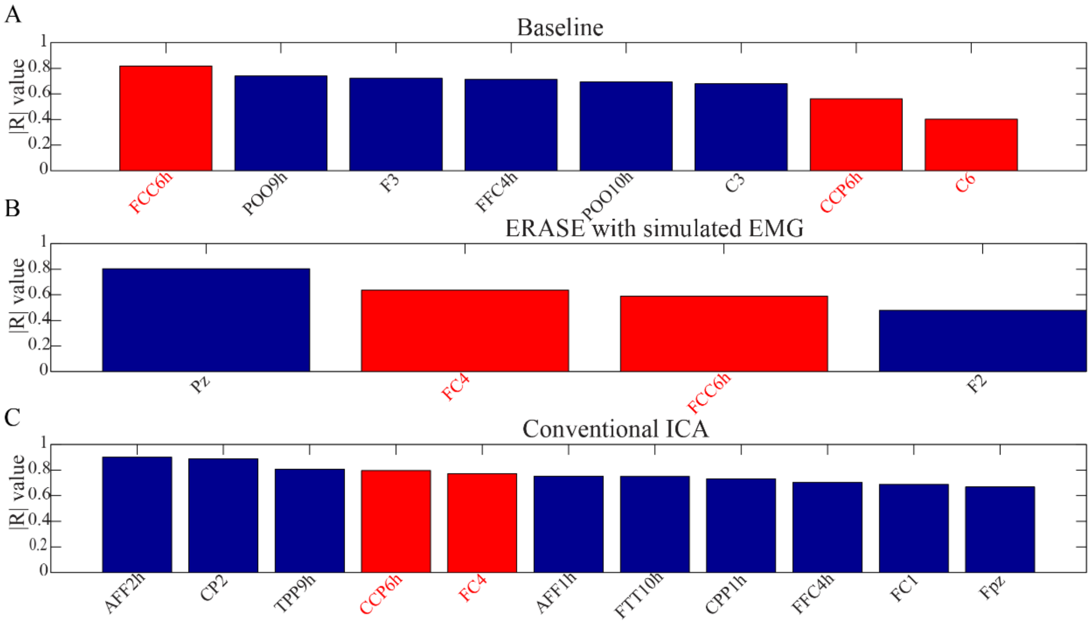

Fig. 9. Bar graphs for showing the electrodes with significant correlation in three conditions (baseline, after ERASE with simulated EMG and after conventional ICA). Here, the correlation coefficients were absolute values from 0 to 1. Data were from Subject 5. Blue bars denoted the

electrodes with significant correlation in the NHAs, and red bars were the ones in HAs. A. the electrodes with significant correlation before ERASE. B. the electrodes with significant correlation after ERASE with simulated EMG. C. the electrodes with significant correlation after conventional ICA.

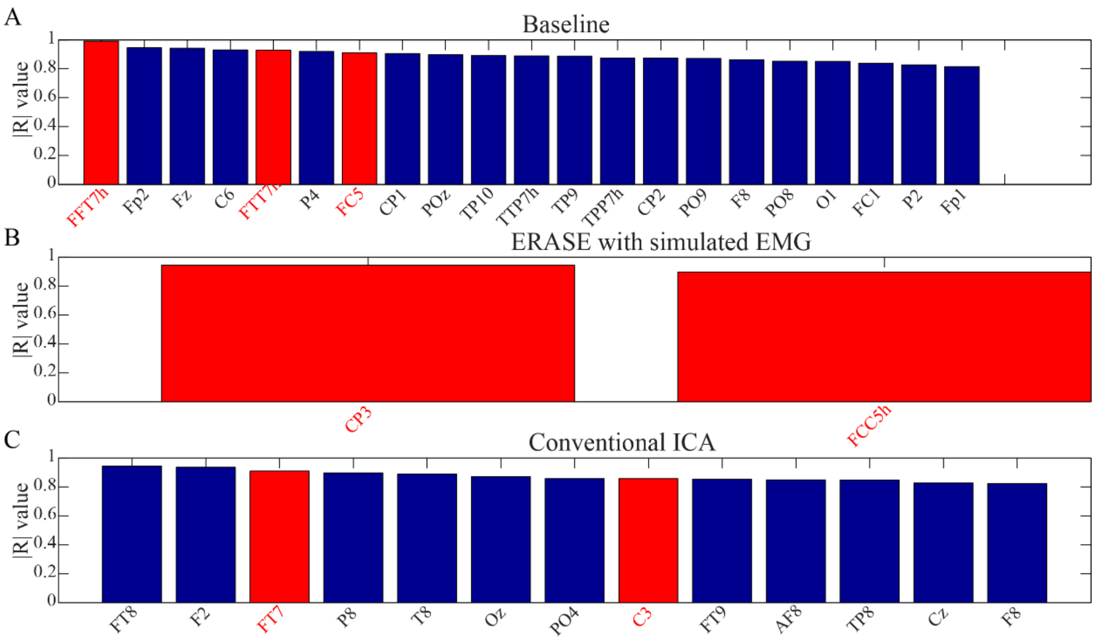

Fig. 10. Bar graphs for showing the electrodes with significant correlation in three conditions (baseline, after ERASE with simulated EMG and after conventional ICA). Here, the correlation coefficients were absolute values from 0 to 1. Data were from Subject 6. Blue bars denoted the electrodes with significant correlation in the NHAs, and red bars were the ones in HAs. A. the electrodes with significant correlation before ERASE. B. the electrodes with significant correlation after ERASE with simulated EMG. C. the electrodes with significant correlation after conventional ICA.

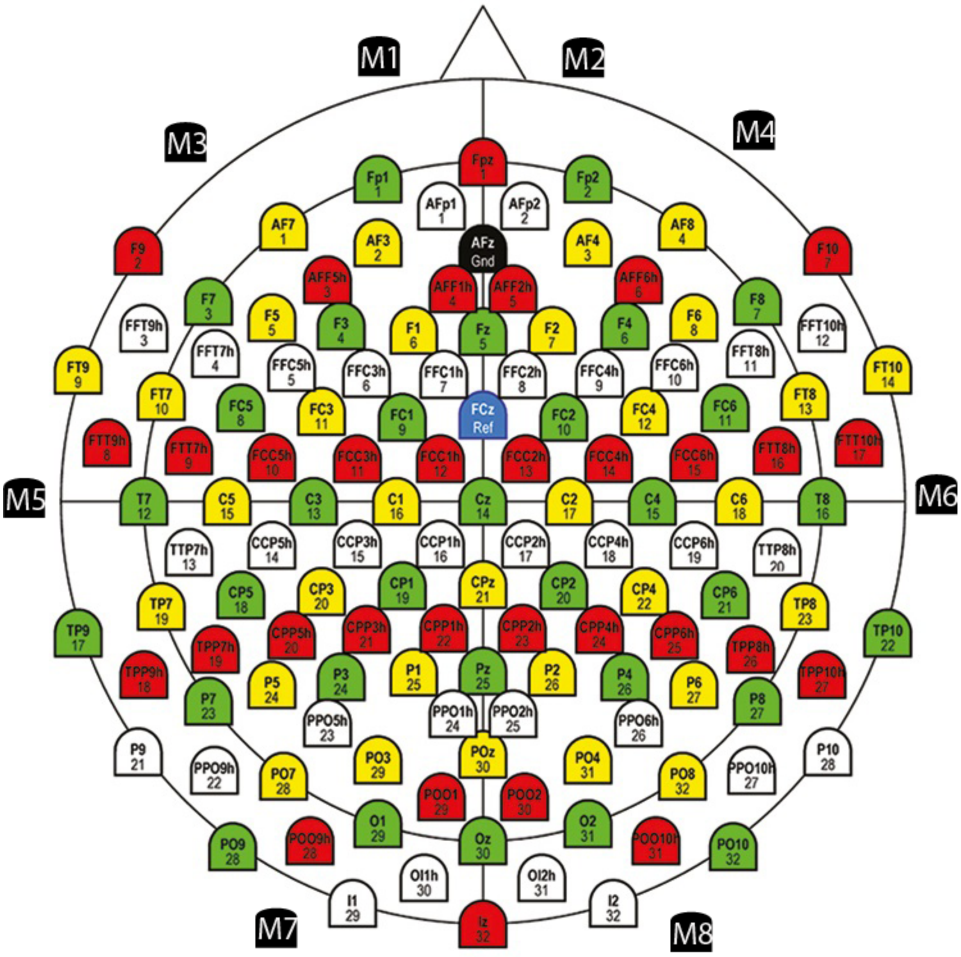

Fig. 11. The 2D image of the electrode locations for the ActiCap EEG cap (Brain Products). M1-M8 denote the locations of EMG signals. M1-M2 denote the locations of bilateral frontalis, M3-M4 denote the locations of bilateral temporalis, M5-M6 denote the locations of bilateral masseter, and M7-M8 denote the locations of bilateral trapezius.
