# Supplementary material for: Refinement of High-Gamma EEG Features From TBI Patients With Hemicraniectomy Using an ICA Informed by Simulated Myoelectric Artifacts
Source: Front Neurosci. 2020 Nov 24;14:599010. doi: 10.3389/fnins.2020.599010 (PMC7732541; doi:10.3389/fnins.2020.599010)
Supplement: Supplementary file 2 — The Appendix includes the contents about the z-scored power of μ and high-γ in different conditions (baseline, after ERASE, and after conventional ICA) for Subject 2–6 (Supplementary Figures 1–5), electrodes with significant correlation in different conditions for Subjects 2–6 (Supplementary Figures 6–10), and the 2D image of the electrode locations (Supplementary Figure 11). [file Data_Sheet_2.ZIP › Appendix.docx]

Part 1. Supplementary results


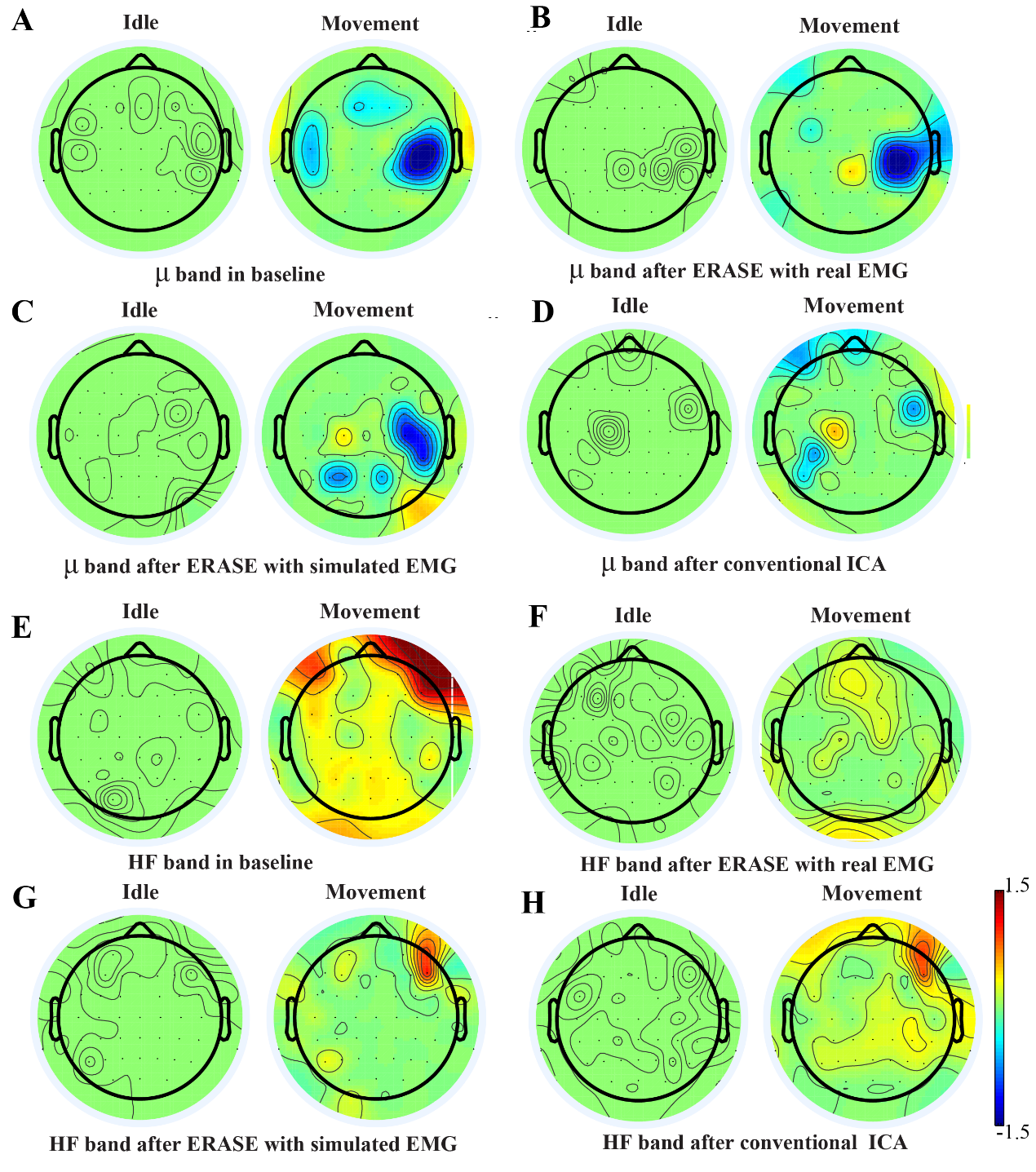


Fig. 1. Brain topography maps for Subject 2 displaying the power of µ band (8 to 12 Hz) and high frequency band (40 to 100 Hz) in different conditions (baseline, after ERASE with real EMG condition and with simulated EMG and after conventional ICA), A-D for µ band and E-H for high frequency band) on the Subject 2. Channels whose z-scored power of µ/high frequency band were not significantly different between idle and movement states (Wilcoxon rank sum test) were nulled (P<0.01 for µ band and 0.05 for high frequency band). The color bar is from -1.5 to 1.5.


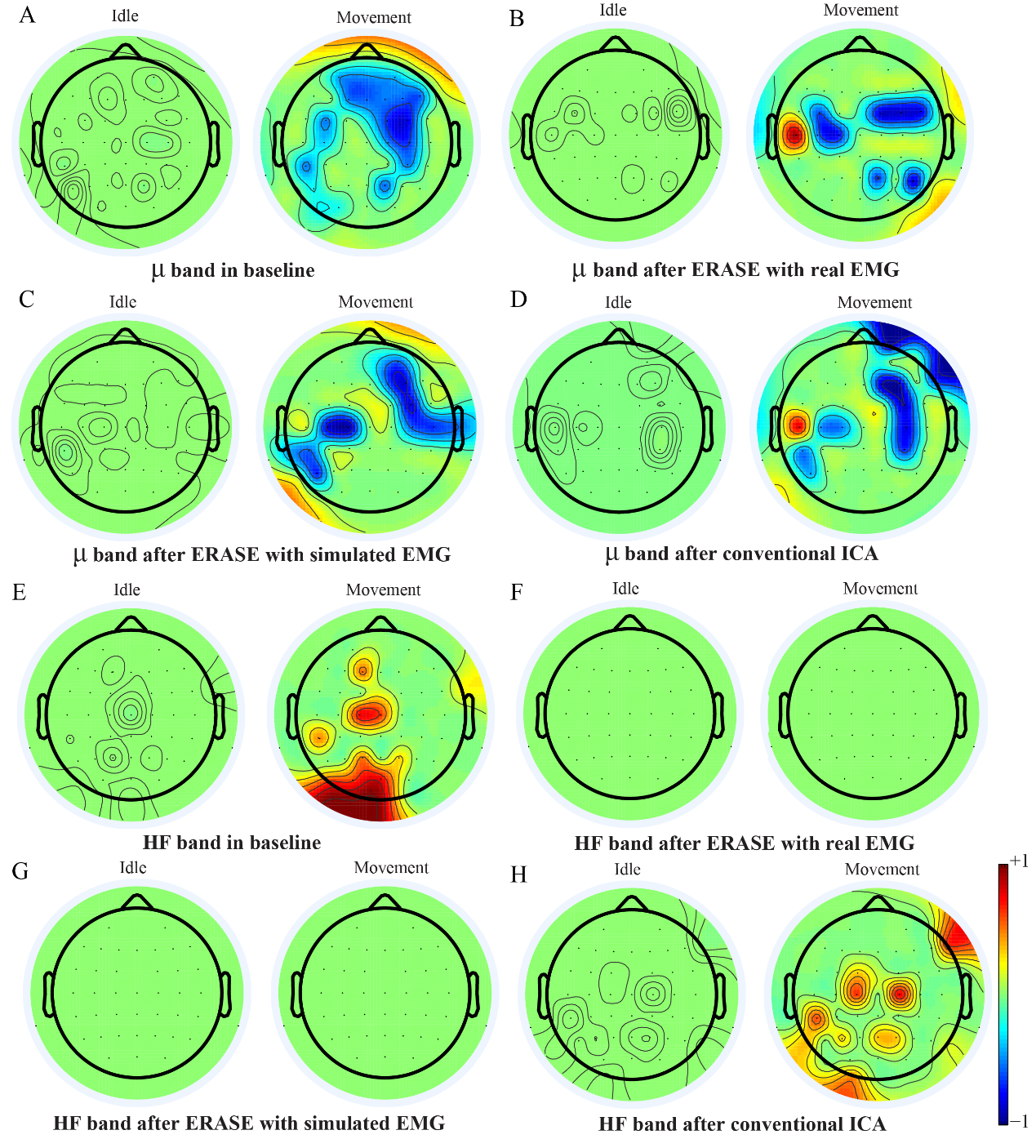


Fig. 2. Brain topography maps for Subject 3 displaying the power of µ band (8 to 12 Hz) and high frequency band (40 to 100 Hz) in different conditions (baseline, after ERASE with real EMG condition and with simulated EMG and after conventional ICA), A-D for µ band and E-H for high frequency band) on the Subject 3. Channels whose z-scored power of µ/high frequency band were not significantly different between idle and movement states (Wilcoxon rank sum test) were nulled (P<0.05 for µ band and 0.05 for high frequency band). The color bar is from -1 to 1.


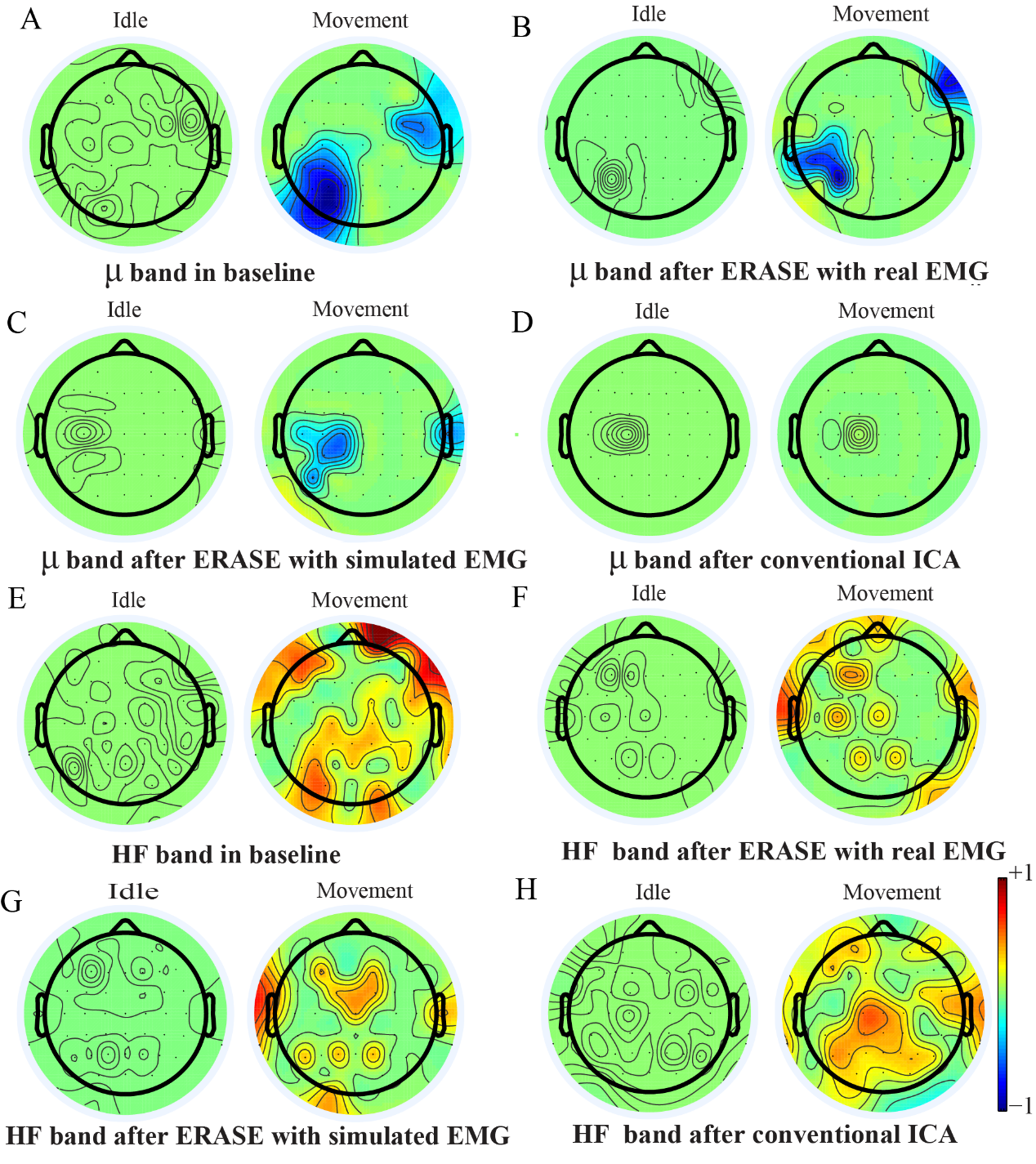


Fig. 3. Brain topography maps for Subject 4 displaying the power of µ band (8 to 12 Hz) and high frequency band (40 to 100 Hz) in different conditions (baseline, after ERASE with real EMG condition and with simulated EMG and after conventional ICA), A-D for µ band and E-H for high frequency band) on the Subject 4. Channels whose z-scored power of µ/high frequency band were not significantly different between idle and movement states (Wilcoxon rank sum test) were nulled (P<0.05 for µ band and 0.05 for high frequency band). The color bar is from -1 to 1.


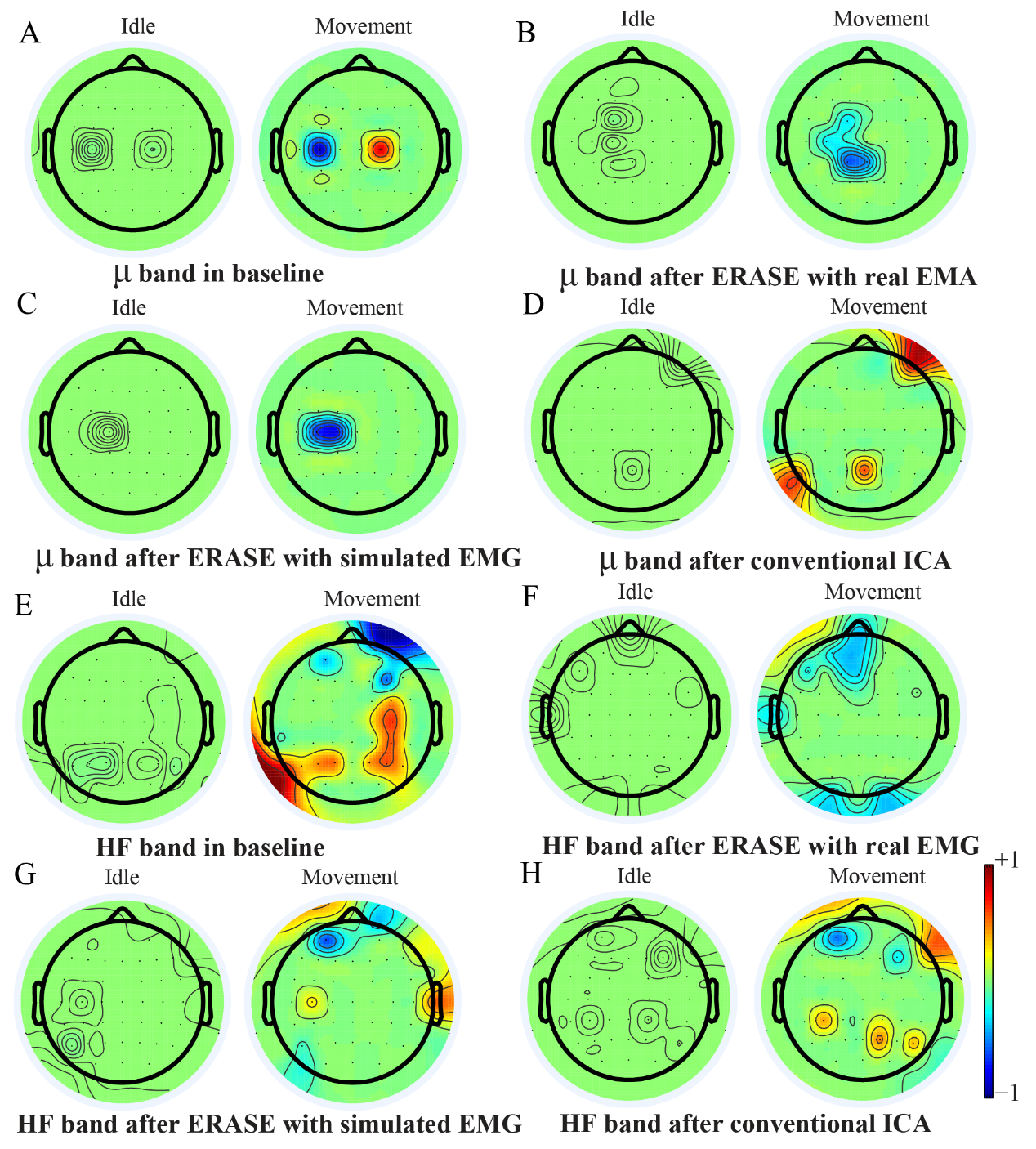


Fig. 4. Brain topography maps for Subject 5 displaying the power of µ band (8 to 12 Hz) and high frequency band (40 to 100 Hz) in different conditions (baseline, after ERASE with real EMG condition and with simulated EMG and after conventional ICA), A-D for µ band and E-H for high frequency band) on the Subject 5. Channels whose z-scored power of µ/high frequency band were not significantly different between idle and movement states (Wilcoxon rank sum test) were nulled (P<0.01 for µ band and 0.05 for high frequency band). The color bar is from -1 to 1.


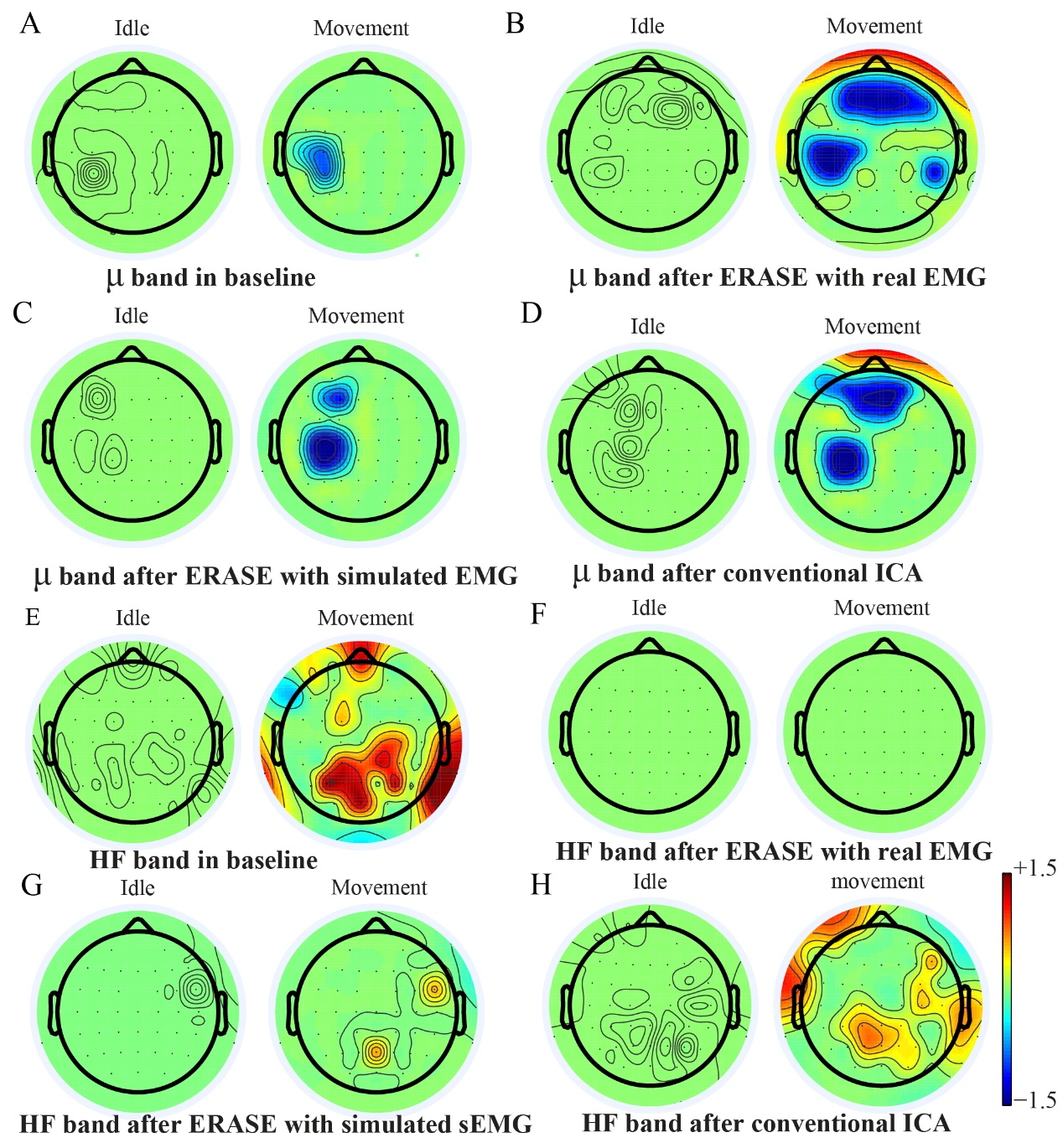


Fig. 5. Brain topography maps for Subject 6 displaying the power of µ band (8 to 12 Hz) and high frequency band (40 to 100 Hz) in different conditions (baseline, after ERASE with real EMG condition and with simulated EMG and after conventional ICA), A-D for µ band and E-H for high frequency band) on the Subject 6. Channels whose z-scored power of µ/high frequency band were not significantly different between idle and movement states (Wilcoxon rank sum test) were nulled (P<0.01 for µ band and 0.05 for high frequency band). The color bar is from -1.5 to 1.5.


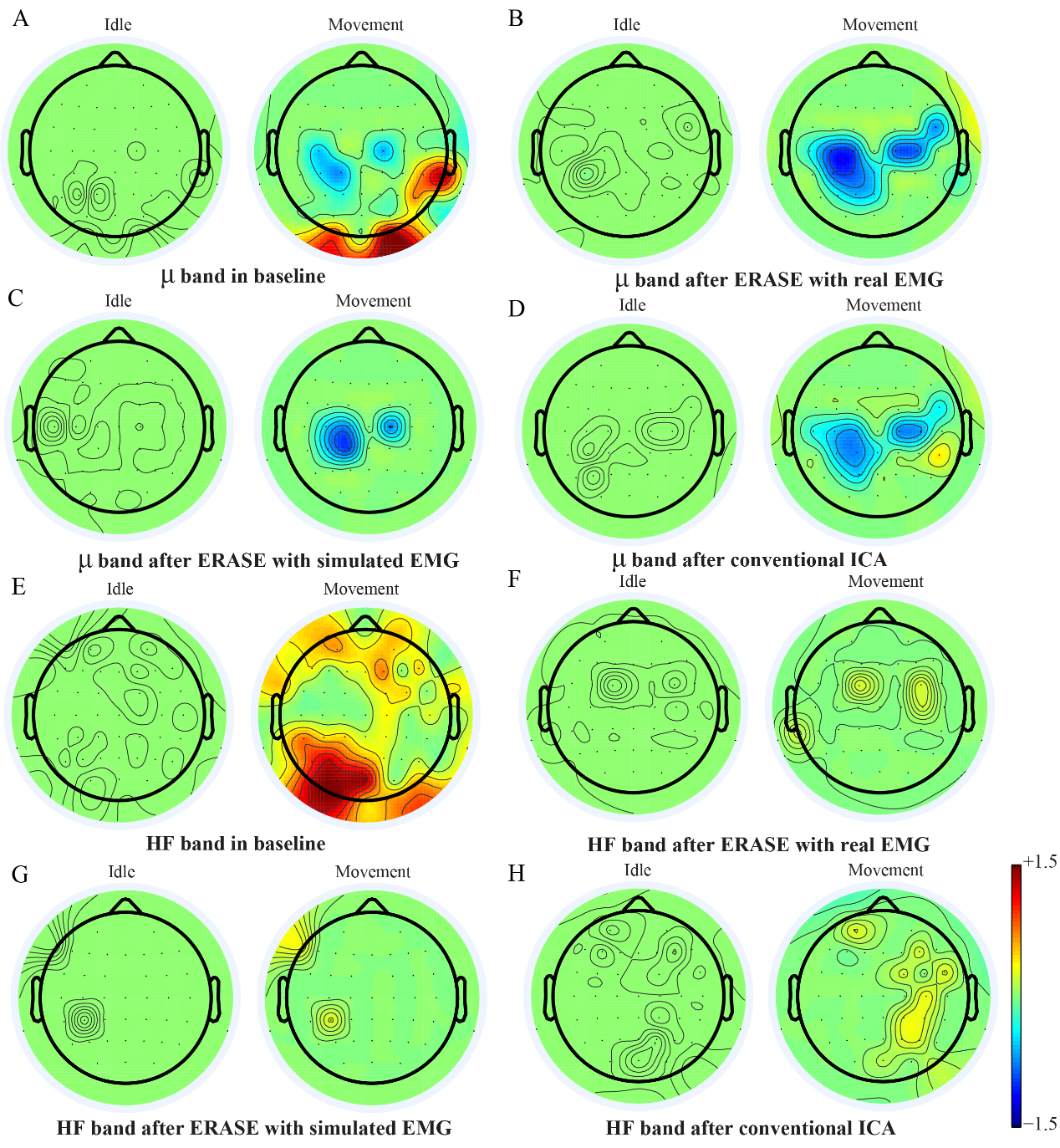


Fig. 6. Brain topography maps for Subject 7 displaying the power of µ band (8 to 12 Hz) and high frequency band (40 to 100 Hz) in different conditions (baseline, after ERASE with real EMG condition and with simulated EMG and after conventional ICA), A-D for µ band and E-H for high frequency band) on the Subject 7. Channels whose z-scored power of µ/high frequency band were not significantly different between idle and movement states (Wilcoxon rank sum test) were nulled (P<0.05 for µ band and 0.05 for high frequency band). The color bar is from -1.5 to 1.5.


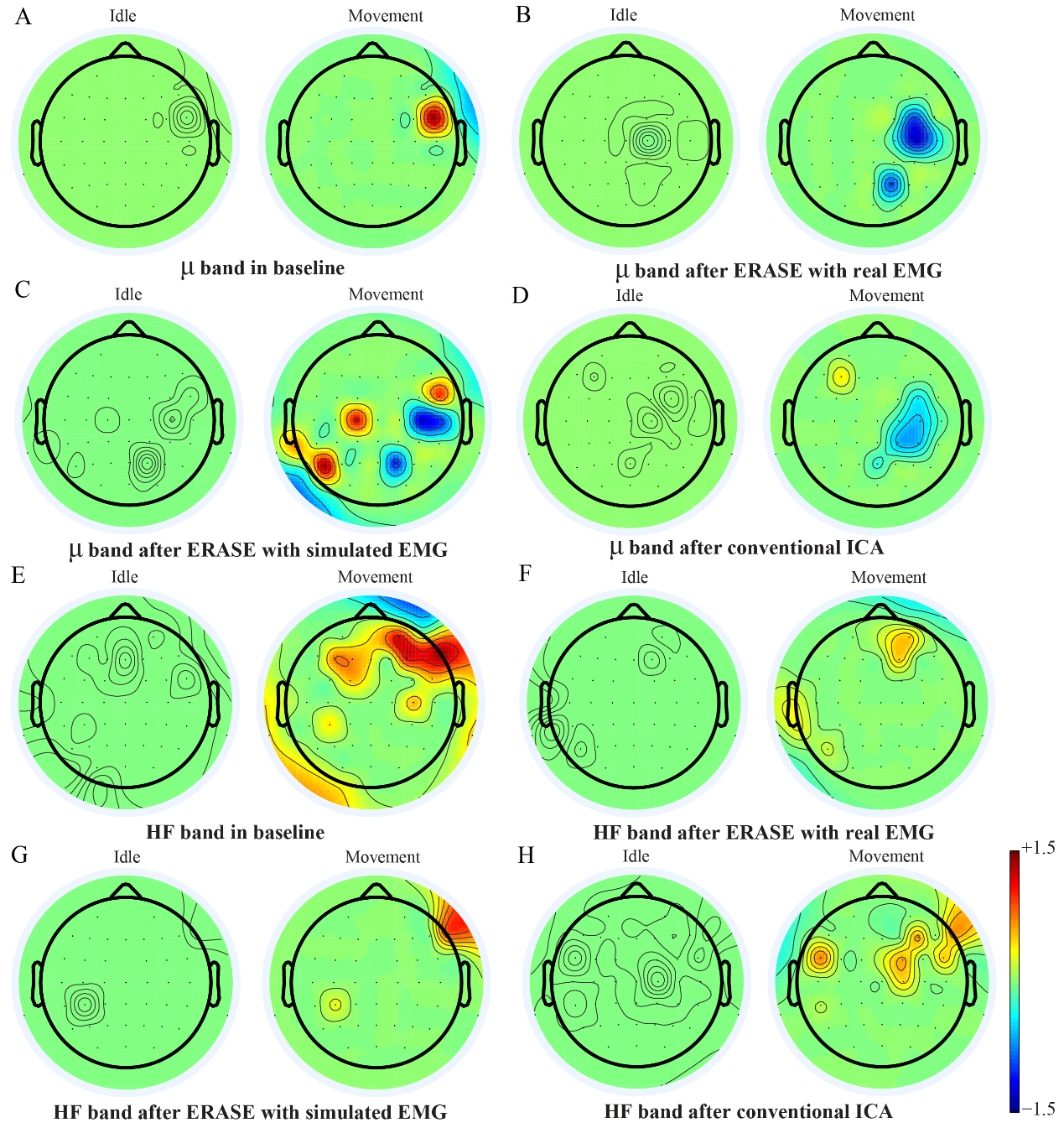


Fig. 7. Brain topography maps for Subject 8 displaying the power of µ band (8 to 12 Hz) and high frequency band (40 to 100 Hz) in different conditions (baseline, after ERASE with real EMG condition and with simulated EMG and after conventional ICA), A-D for µ band and E-H for high frequency band) on the Subject 8. Channels whose z-scored power of µ/high frequency band were not significantly different between idle and movement states (Wilcoxon rank sum test) were nulled (P<0.05 for µ band and 0.05 for high frequency band). The color bar is from -1.5 to 1.5.


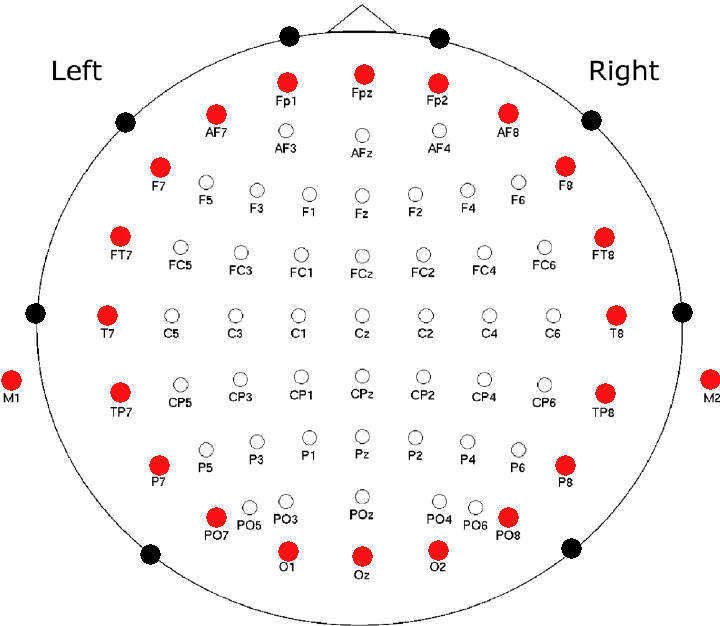


Figure 8. electrodes location of 64-channel cap. The red dots denote the hat band electrodes. The black dots outline the position of the added EMG channels.


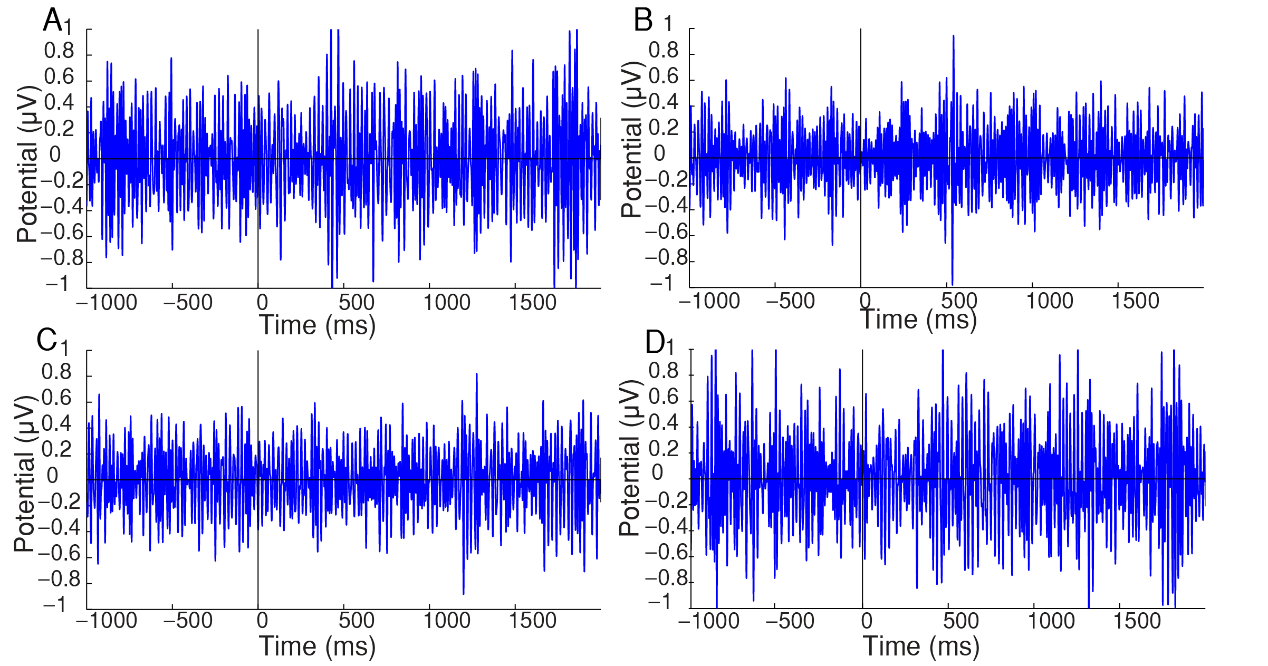


Figure 9. Time series band-pass filtered with frequency band from 40 Hz to 100Hz in different conditions (baseline, after ERASE with real EMG condition and with simulated EMG and after conventional ICA). Here, we show the data in one trial from C6 electrode. Data is from Subject 1. A. Time series baseline. B. Time series after ERASE with real EMG. C. Time series after ERASE with simulated EMG. D. Time series after conventional ICA.


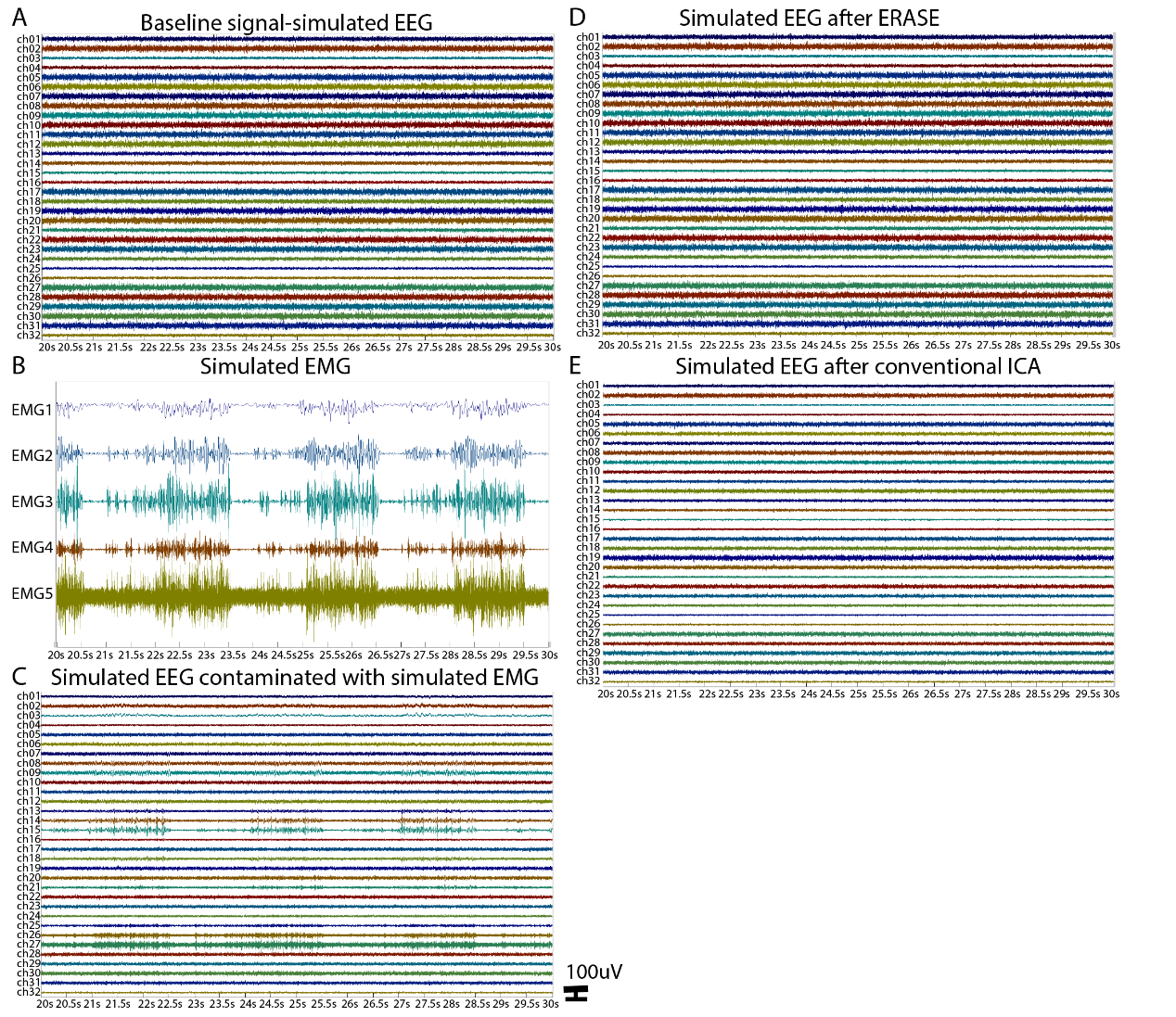


Figure 10. Sample of 10s time series. A. simulated EEG. B. simulated EMG. C. contaminated EEG signal (simulated EEG was contaminated by simulated EMG). In this example, SNR is 1. D. recovered EEG after running ERASE. E. recovered EEG after running conventional ICA.


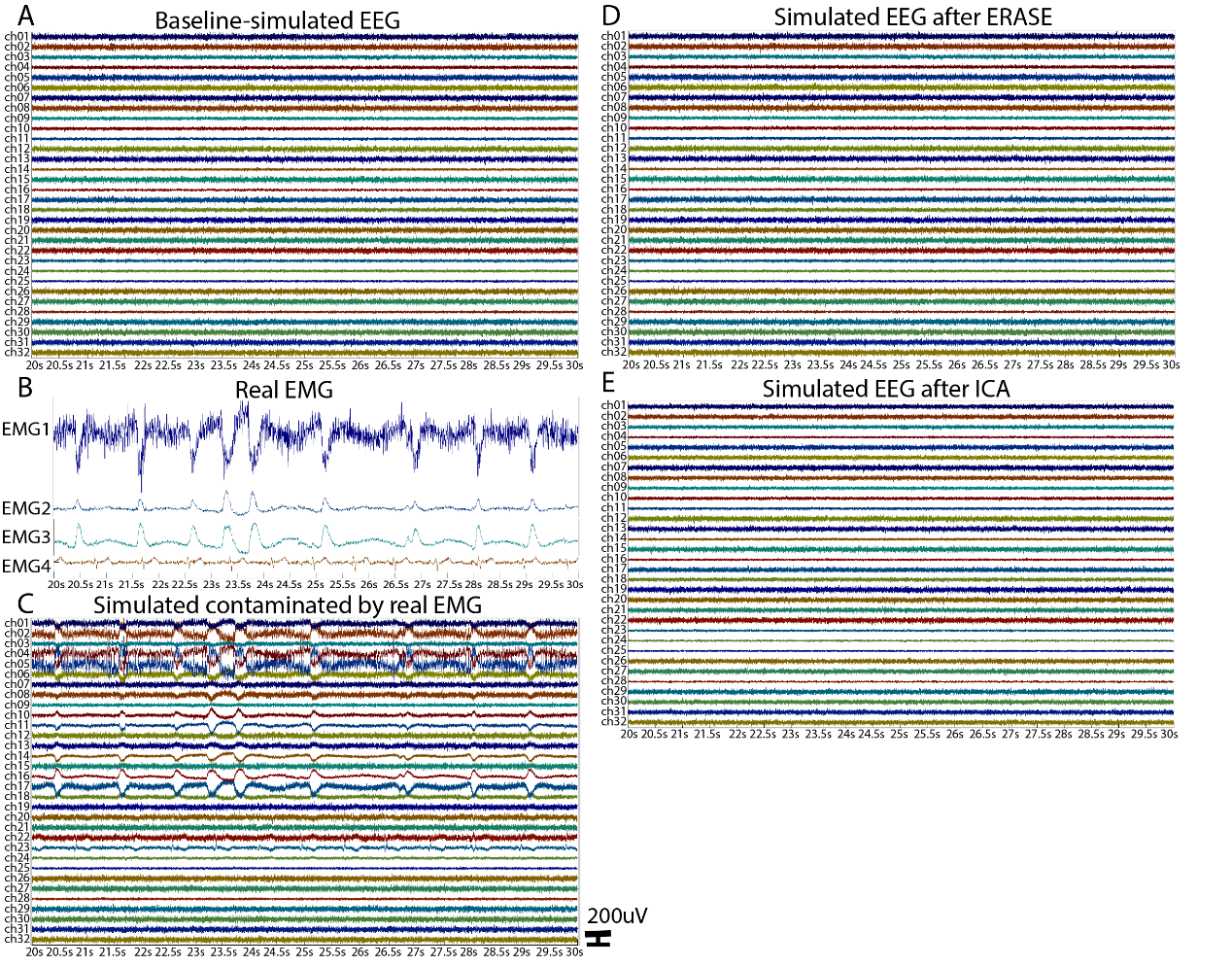


Figure 11. Sample of 10s time series. A. simulated EEG. B. real EMG. All real EMG in this figure was recorded from healthy subject (Subject 1). C. contaminated EEG signal (simulated EEG was contaminated by real EMG). In this example, SNR is 1. D. recovered EEG after running ERASE. E. recovered EEG after running conventional ICA.


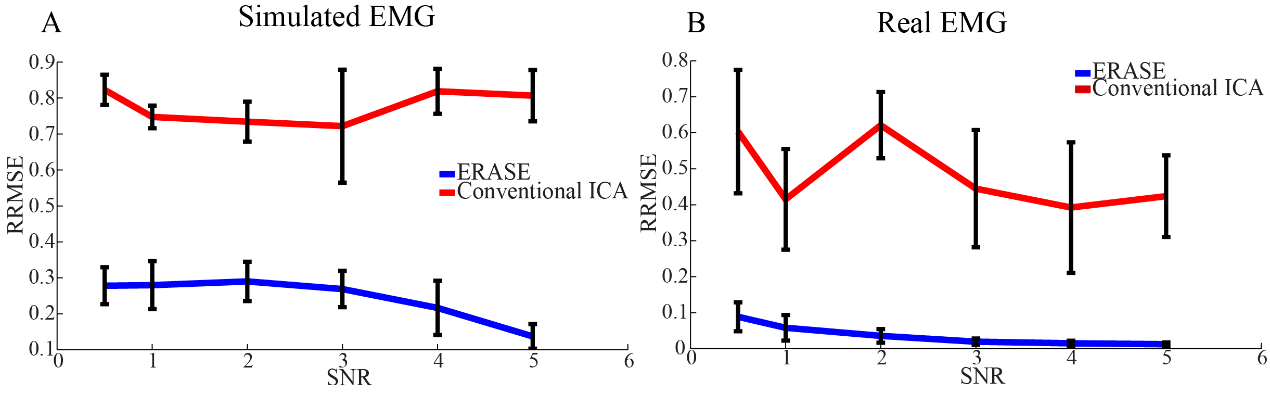


Figure 12. Relative root mean squared error (RRMSE) with varying SNR. A. RRMSE after running ERASE (blue line) and conventional ICA (red line) in the simulated EMG condition (simulated EMG was considered as contaminant). B. RRMSE after running ERASE (blue line) and conventional ICA (red line) in the real EMG condition (real EMG was considered as contaminant). The definition of RRMSE and SNR here can refer to literatures [1,38]. 200 simulated EEG were generated by the method described in Method in manuscript. Each EEG was contaminated by simulated EMG and real EMG, respectively, with different SNRs (0.5, 1, 2, 3, 4, 5). Each contaminated EEG was subjected to ERASE and conventional ICA, respectively, and artifacts-free EEG data can be obtained. RRMSE was calculated for each artifacts-free EEG. Mean and standard deviation of RRMSE were calculated across those data with same SNR.

Table 1. Average false positive threshold for two variables

| Number of contaminated EEG electrodes | | | | | |
| --- | --- | --- | --- | --- | --- |
| Parameter value | 6 | 12 | 18 | 24 | 30 |
| Average threshold | 0.2886 $\pm$ 0.0034 | 0.2886 $\pm$ 0.0035 | 0.278 $\pm$ 0.0031 | 0.2885 $\pm$ 0.0033 | 0.2886 $\pm$ 0.0035 |
| Number of added EMG channels | | | | | |
| Parameter value | 1 | 2 | 3 | 4 | 5 |
| Average threshold | 0.2887 $\pm$ 0 | 0.2886 $\pm$ 0.0031 | 0.2885 $\pm$ 0.0033 | 0.2886 $\pm$ 0.0032 & | 0.2887 $\pm$ 0.0035 |

mean $\pm$ S.D.

The threshold was defined as 5% of the maximal noise coefficient in the noise ICs column.

For the test of contaminated EEG electrodes, three types of EMG (simulated EMG signals from frontalis, temporalis, and posterior head muscles) were used.

For the test of added EMG channels, each type of EMG was employed to contaminate 6 EEG electrodes.

Table 2. Average sensitivity threshold for two variables

| Number of contaminated EEG electrodes | | | | | |
| --- | --- | --- | --- | --- | --- |
| Parameter value | 6 | 12 | 18 | 24 | 30 |
| Average threshold | 0.3664 $\pm$ 0.0031 | 0.3665 $\pm$ 0.0031 | 0.3664 $\pm$ 0.0032 | 0.3663 $\pm$ 0.003 | 0.3663 $\pm$ 0.0028 |
| Number of added EMG channels | | | | | |
| Parameter value | 1 | 2 | 3 | 4 | 5 |
| Average threshold | 0.242 $\pm$ 0 | 0.2864 $\pm$ 0.0001 | 0.3274 $\pm$ 0.0005 | 0.3896 $\pm$ 0.00032 | 0.4583 $\pm$ 0.00052 |

mean $\pm$ S.D.

The threshold was defined as 5% of the maximal noise coefficient in the noise ICs column.

For the test of contaminated EEG electrodes, three types of EMG (simulated EMG signals from frontalis, temporalis, and posterior head muscles) were used.

For the test of added EMG channels, each type of EMG was employed to contaminate 6 EEG electrodes.

Table 3. Average noise index for two scenarios

| Number of contaminated EEG electrodes | | | | | |
| --- | --- | --- | --- | --- | --- |
| Parameter value | 6 | 12 | 18 | 24 | 30 |
| Average noise index (After ERASE with simulated EMG) | 92.0419 $\pm$ 12.084 | 54.9098 $\pm$ 7.6664 | 40.2554 $\pm$ 6.0737 | 31.3284 $\pm$ 4.8454 | 25.3174 $\pm$ 3.8913 |
| Average noise index (conventional ICA) | 10.5437 $\pm$ 7.9094 | 5.8600 $\pm$ 3.7891 | 3.8574 $\pm$ 2.4690 | 3.1893 $\pm$ 1.8570 | 2.4541 $\pm$ 1.1627 |
| Number of added EMG channels | | | | | |
| Parameter value | 1 | 2 | 3 | 4 | 5 |
| Average noise index (After ERASE with simulated EMG) | 33.4363 $\pm$ 6.9448 | 39.8032 $\pm$ 6.2961 | 37.7042 $\pm$ 7.9054 | 32.5832 $\pm$ 5.2438 | 39.7336 $\pm$ 9.6851 |
| Average noise index (conventional ICA) | 26.0746 $\pm$ 11.839 | 28.4582 $\pm$ 12.895 | 24.8897 $\pm$ 12.24 | 25.4884 $\pm$ 8.9587 | 27.7924 $\pm$ 8.8771 |

mean $\pm$ S.D.
